# Malignant Hypercalcemia Due to the Ectopic Production of Calcitriol by an Abdominal Liposarcoma

**DOI:** 10.7759/cureus.33446

**Published:** 2023-01-06

**Authors:** Ana Figueiredo, Teresa Pereira, Rafael Cabrera, Joana Simões-Pereira, Valeriano Leite

**Affiliations:** 1 Endocrinology, Instituto Português de Oncologia de Lisboa Francisco Gentil, Lisboa, PRT; 2 Pathology, Instituto Português de Oncologia de Lisboa Francisco Gentil, Lisboa, PRT

**Keywords:** immunohistochemistry (ihc), 1α-hydroxylase, dedifferentiated liposarcoma, calcitriol, malignant hypercalcemia

## Abstract

Hypercalcemia of malignancy (HM) is a common form of paraneoplastic syndrome associated with a poor prognosis of the disease. In solid tumors, HM occurs mainly due to the production of parathyroid hormone-related peptide (PTHrP). We present a case of a 60-year-old male with a 25 cm retroperitoneal liposarcoma diagnosed with severe hypercalcemia (16.8 mg/dL) by a preoperative blood sampling. Hypercalcemia workup showed suppressed parathyroid hormone (PTH), normal PTHrP, and high 1,25-dihydroxyvitamin D (1,25(OH)2D) serum levels. After surgery, hypercalcemia and calcitriol levels normalized. Immunohistochemical analysis of the tumor showed 1α-hydroxylase expression by tumor cells. To our knowledge, this is the first case of liposarcoma-associated hypercalcemia caused exclusively by the ectopic production of calcitriol. Despite being a rare cause of hypercalcemia, measuring 1,25(OH)2D should be considered in the workup of a patient with high serum calcium levels, suppressed PTH, and normal PTHrP.

## Introduction

Hypercalcemia occurs in up to 30% of patients with malignancy, being associated with advanced disease and poor prognosis [1]. It is one of the most common forms of paraneoplastic syndromes, with an incidence of 15 cases per 100,000 people per year [2]. Hypercalcemia of malignancy (HM) occurs in many types of cancer, with higher frequency in breast cancer, lung cancer, and multiple myeloma [3]. There are several mechanisms responsible for HM, which include increased secretion of parathyroid hormone-related peptide (PTHrP), local osteolytic hypercalcemia, excess extrarenal production of 1,25-dihydroxyvitamin D (1,25 (OH)2D), and primary or ectopic secretion of parathyroid hormone (PTH) [1]. PTHrP-mediated hypercalcemia accounts for 80% of HM cases and is mainly associated with squamous cell carcinomas of the lung, head and neck, and esophagus or with breast, kidney, prostate, and bladder cancers, but virtually, any tumor can cause this syndrome [4]. The secretion of 1,25(OH)2D accounts for approximately 1% of cases of HM [1]. It is primarily associated with hematologic malignancies such as Hodgkin and non-Hodgkin lymphoma but has also been reported in some solid tumors such as ovarian dysgerminoma, gastrointestinal stromal tumor (GIST), and seminomas [1,5]. Here, we present a patient with abdominal liposarcoma and HM caused by the production of 1,25(OH)2D.

## Case presentation

**Figure 1:**
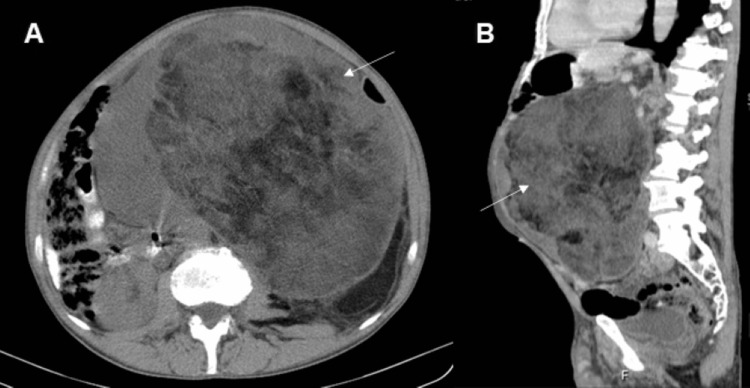
Computed tomography scan of the abdomen and pelvis showing an 18 × 25 cm left retroperitoneal mass: axial (A) and sagittal (B) view (white arrow).

A 60-year-old male, with no relevant medical history, was diagnosed in June 2019 with a 25 cm retroperitoneal mass following a progressive increase in scrotal and abdominal volume over a period of six months (Figure 1). A computed tomography (CT)-guided biopsy was suggestive of liposarcoma, and surgery was proposed.

Preoperative blood tests showed serum calcium of 16.8 mg/dL (normal range (NR): 8.4-10.2 mg/dL), which led to an urgent referral to our department. He started intensive fluid replacement, loop diuretics, and glucocorticoids. Electrocardiogram was normal. Therapy with bisphosphonate was not performed since the estimated glomerular filtration rate (eGFR) was 21.7 mL/minute/1.73 m^2^. He reported nonspecific symptoms such as fatigue, myalgia, anorexia, and mood changes in the last few weeks. Drug-induced hypercalcemia was excluded as he denied intake of any medication prior to admission. The initial investigation showed albumin-corrected calcium level of 16.8 mg/dL, phosphorous of 6.3 mg/dL (NR: 2.3-4.7 mg/dL), PTH < 3 pg/mL (NR: 12-65 pg/mL), 25-hydroxyvitamin D (25(OH)D) of 18 ng/mL (NR: 8-56 ng/mL), and alkaline phosphatase of 91 UI/L (NR: 40-150 UI/L) (Table 1). Bone scan scintigraphy was negative, and multiple myeloma was excluded by serum and urine protein electrophoresis. At this time, serum PTHrP and 1,25(OH)2D levels were requested, but the results were not available until a few weeks later. After five days, he was discharged from the hospital with a serum calcium of 12.3 mg/dL, clinically asymptomatic, and with the recommendation to increase oral fluid intake.

**Table 1:** Laboratory investigations for the workup of hypercalcemia. eGFR, estimated glomerular filtration rate; PTHrP, parathyroid hormone-related peptide; 25(OH)D, 25-hydroxyvitamin D; 1,25(OH)2D, 1,25-dihydroxyvitamin D

Laboratory test	First hospital admission	Recurrence	Reference range
Albumin-corrected calcium (mg/dL)	16.8	11.9	8.4-10.2
Phosphorous (mg/dL)	6.3	3.8	2.3-4.7
eGFR (mL/minute/1.73 m^2^)	21.7	49	>60
PTH (pg/mL)	<3	<3	12-65
PTHrP (pmol/L)	0.9	1.2	<2.5
25(OH)D (ng/mL)	18	34	8-56
1,25(OH)2D (pg/mL)	224	169	18-71

**Figure 2:**
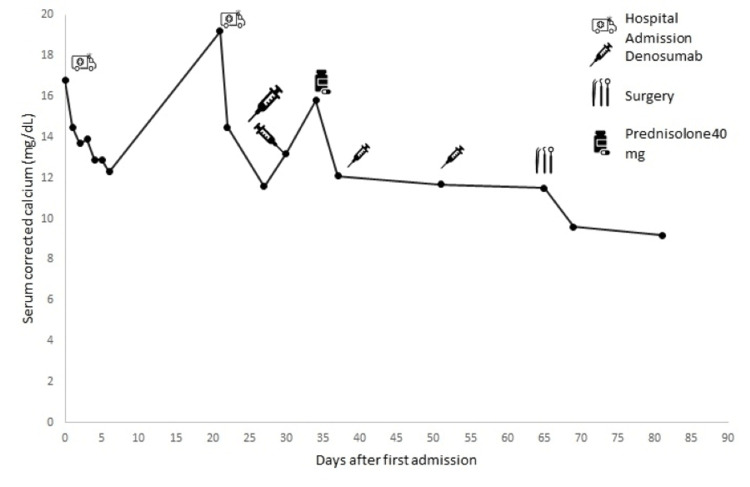
Levels of albumin-corrected serum calcium and response to therapy.

Two weeks later, he was readmitted because of a corrected calcium level of 19.2mg/dL. At this time, he complained of fatigue, musculoskeletal pain, anorexia, and constipation. The electrocardiographic evaluation was normal. Since he maintained an eGFR < 30 mL/minute/1.73 m^2^, denosumab 120 mg was administered subcutaneously on days 1, 8, 15, and 29 and then every four weeks. After four days of the first administration, serum calcium decreased to 11.6 mg/dL, and the patient was discharged from the hospital. He maintained a close follow-up at our clinic, with serum calcium levels between 11.6 mg/dL and 15.8 mg/dL (Figure 2). This last value occurred between the second and the third administration of denosumab, and at this time, the patient was given prednisolone 40 mg orally for three days, until the next administration of denosumab, and calcium levels dropped to 12.1 mg/dL. The results of the initial workup became available, showing PTHrP of 0.9 pmol/L (NR: PTHrP < 2.5 pmol/L) and a substantially increased 1,25 (OH)2D serum levels at 224 pg/mL (NR: 18-71 pg/mL).

**Figure 3:**
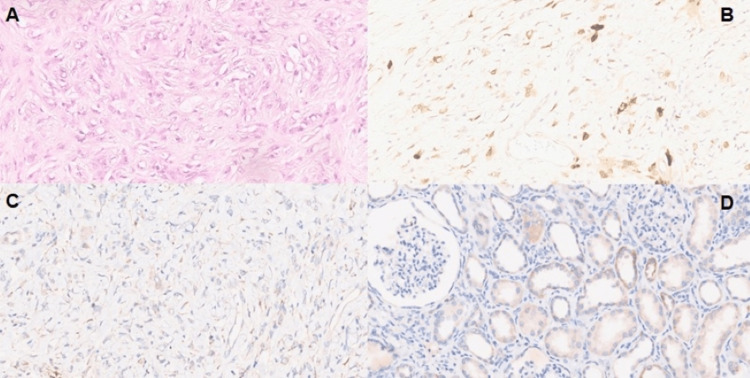
(A) Dedifferentiated area of liposarcoma (hematoxylin & eosin stain). (B) MDM2 positivity in the dedifferentiated area of liposarcoma. (C) Weak positivity for 1α-hydroxylase expression in liposarcoma tissue. (D) Positivity for 1α-hydroxylase expression in normal human kidney.

Surgical resection of the tumor was performed in September 2019, and pathology revealed a dedifferentiated liposarcoma measuring 45 × 40 × 29 cm (Figure 3A). Immediately after the surgical intervention, serum calcium and 1,25(OH)2D levels dropped to normal (8.66 mg/dL and 54 pg/mL, respectively). Staging after surgery showed a complete resection, and at follow-up, there was no evidence of disease until 33 months later (almost three years), when hypercalcemia returned (11.9 mg/dL), and a CT scan showed local recurrence of the tumor. Further investigation showed phosphorus of 3.8 mg/dL, PTH < 3 pg/mL, 25(OH)D of 34 ng/mL, 1,25(OH)2D of 169 pg/mL, and PTHrP < 0.5 pmol/L. Zoledronic acid 3.3 mg (dose adjusted to eGFR of 49 mL/minute) was administered intravenously, with improvement in serum calcium levels (9.9 mg/dL) seven days after treatment. Intravenous doxorubicin 75 mg/m^2^ was initiated in monotherapy, every 21 days, but was discontinued after five cycles, at the end of July, because of disease progression. Serum calcium levels have remained within the normal range, but the second administration of zoledronic acid was needed in August, due to serum calcium of 12.5 mg/dL. Second-line therapy with trabectedin 1.5 mg/m^2^ is now under consideration.

Immunohistochemistry for 1α-hydroxylase

Tumor tissue sections were stained with antiserum raised against rabbit renal 1α-hydroxylase by Ventana BenchMark ULTRA IHC system (Roche Diagnostics, Basel, Switzerland). The primary antibodies anti-CYP27B1 (polyclonal reference PA5-26065, 1:15/28 minutes) (Thermo Fisher Scientific, Waltham, MA, USA) and anti-MDM2 (reference MAD-000682QD-7, PD/16 minutes) (Vitro Master Diagnostic Mouse Monoclonal IF2) were used with the peroxidase-indirect-polymer method Ventana Optiview DAB refª 760-700. Sections 3 μm thick were cut unto Superfrost plus slides from paraffin-embedded routine tissue blocks. The heat-mediated antigen retrieval was Ventana CC1 for 64 minutes for anti-CYP27B1 and 48 minutes for anti-MDM2 (Figure 3B). For positive and negative controls, a kidney and a tonsil were used, respectively. Immunohistochemical analysis with the antiserum for 1α-hydroxylase showed expression for the enzyme in liposarcoma cells (Figure 3C) and in renal proximal tubule epithelial cells (Figure 3D). By contrast, no staining for 1α-hydroxylase was observed in tonsil tissue (data not shown).

## Discussion

Herein, we describe a case of a patient with a dedifferentiated liposarcoma and HM associated with exclusive production of 1,25(OH)2D. Most cases of HM are caused either by the secretion of PTHrP or by osteolytic bone metastasis [3]. The ectopic production of 1,25(OH)2D is an uncommon cause of HM and can occur in sarcoidosis and in other nonmalignant granulomatosis diseases, as well as in malignancy, such as in Hodgkin and non-Hodgkin lymphomas [3,4]. There are few cases reported in the literature of HM associated with sarcomas [6-9]. The production of 1,25(OH)2D as the cause of HM was reported in one case of dedifferentiated abdominal liposarcoma, in which there was a coproduction of both 1,25(OH)2D and PTHrP by the tumor [10].

In our case study, the definitive treatment of hypercalcemia was the surgical resection of the tumor. Until surgical intervention, severe hypercalcemia was managed with denosumab since bisphosphonates are contraindicated in patients with severe renal impairment. Hu et al. showed that denosumab could be used to reduce serum calcium in patients with bisphosphonate-refractory HM [11]. Based on this study, we apply the same therapeutic scheme, with the improvement of calcium levels after four days of denosumab initiation. However, between administrations, serum calcium levels tended to rise, being notorious for an improvement with the addition of prednisolone, which retrospectively favors calcitriol ectopic production.

The exact mechanism of the production of 1,25(OH)2D in HM is unknown and has been studied. In our patient, the definitive resolution of hypercalcemia after surgical resection favors the hypothesis that the tumor harbored the cells responsible for the calcitriol-mediated hypercalcemia, and the immunohistochemical expression of 1α-hydroxylase in tumor cells confirms it (Figure 3C). Hewison et al. reported a case of a 75-year-old male with hypercalcemia and Hodgkin lymphoma who was submitted to splenectomy with the resolution of hypercalcemia [12]. Immunohistochemical analysis of splenic tissue sections showed negative expression for 1α-hydroxylase in lymphoma and in normal spleen cells but positivity in the surrounding macrophages. Evans et al. also studied the expression of 1α-hydroxylase in a collection of 12 dysgerminomas by RT-PCR analysis, enzyme assays, and immunolocalization studies, all of them showing increased expression of this enzyme in both tumor cells and infiltrated macrophages [13]. Although the impact of the expression of 1α-hydroxylase on the tumor is unknown, it is postulated that it may be part of an endogenous tumor defense mechanism, since calcitriol has immunosuppressive effects on cells of the immune system [9,13].

## Conclusions

We presented a unique case of HM associated with calcitriol production by a dedifferentiated liposarcoma that was temporarily resolved by tumor resection. In our patient, hypercalcemia represented an accurate biomarker of tumor recurrence almost three years after primary surgery.

Despite being a rare cause of hypercalcemia, ectopic 1,25(OH)2D production should always be considered in the workup of HM when PTHrP production has already been excluded.
